# Use of surgical glue versus suture to repair perineal tears: a randomised controlled trial

**DOI:** 10.1186/s12884-023-05565-x

**Published:** 2023-04-12

**Authors:** Adriana Caroci-Becker, Wesllanny Sousa Brunelli, Marlise de Oliveira Pimentel Lima, Angela Megumi Ochiai, Sheila Guimarães Oliveira, Maria Luiza Riesco

**Affiliations:** 1 School of Nursing, University of São Paulo, São Paulo, Brazil; 2 School of Arts, Sciences and Humanities, University of São Paulo, São Paulo, Brazil; 3 Municipal Hospital Dr Moses Deustch, São Paulo, Brazil

**Keywords:** Nurse, Midwifery, Perineum, Tears, Sutures, Tissue adhesives, Cyanoacrylates

## Abstract

**Background:**

Surgical glue has been used in several body tissues, including perineal repair, and can benefit women.

**Objectives:**

To evaluate the effectiveness of n-butyl-2-cyanoacrylate surgical glue compared to the polyglactin 910 suture in repairing first- and second-degree perineal tears and episiotomy in vaginal births.

**Design:**

A parallel randomised controlled open trial.

**Setting:**

Birth centre in Itapecerica da Serra, São Paulo, Brazil.

**Participants and methods:**

The participants were 140 postpartum women allocated into four groups: two experimental groups repaired with surgical glue (*n* = 35 women with a first-degree tear; *n* = 35 women with a second-degree tear or episiotomy); two control groups sutured with thread (*n* = 35 women with a first-degree tear; *n* = 35 women with a second-degree tear or episiotomy). The outcomes were perineal pain and the healing process. Data collection was conducted in six stages: (1) up to 2 h after perineal repair; (2) from 12 to 24 h postpartum; (3) from 36 to 48 h; (4) from 10 to 20 days; (5) from 50 to 70 days; and (6) from 6 to 8 months. ANOVA, Student's t, Monte Carlo, x-square and Wald tests were used for the statistical analysis.

**Results:**

One hundred forty women participated in the first three stages, 110 in stage 4, 122 in stage 5, and 54 in stage 6. The women treated with surgical glue had less perineal pain (*p* ≤ 0.001). There was no difference in the healing process, but the CG obtained a better result in the coaptation item (*p *≤ 0.001).

**Conclusions:**

Perineal repair with surgical glue has low pain intensity and results in a healing process similar to suture threads.

**Trial registration:**

Brazilian Registry of Clinical Trials (UTN code: U1111-1184-2507; RBR-2q5wy8o); date of registration 01/25/2018; www.ensaiosclinicos.gov.br/rg/RBR-2q5wy8/

## Introduction

Perineal trauma in vaginal birth can negatively influence women's physical, physiological, psychological and social well-being with short- and long-term consequences [1, 2]. Nearly 70.3% of women present some perineal trauma at delivery, 18.2% present first-degree tears and 40.6% second-degree tears [3]. Nulliparous women present approximately 2.5 times more chances of suffering some perineal trauma at delivery than multiparous women [4].

The literature indicates that perineal pain related to perineal traumas is present in many primiparous women during the first year after birth, reported in one out of ten mothers [5]. The incidence of complications in the healing process resulting from perineal traumas varies between 0.1% and 23.6% due to infection and from 0.2% to 24.6% to dehiscence [6].

Currently, the fast-absorbing polyglycolic suture thread (Vicryl® rapid) with the continuous technique is the primary choice for perineal repair, as it presents better results in pain and perineal healing [7]. However, adhesive glue shows excellent potential for changing the perineal repair technique, as it presents similar or better results to the Vicryl® rapid suture thread [8–10].

One of the first studies that compared the use of fast-absorbing polyglycolic suture with octyl-2-cyanoacrylate surgical glue in the perineal repair of first-degree tears was conducted with 102 women (divided into two groups: 28 sutured women and 74 with glue repair), monitored during six weeks. It concluded that the use of glue presented cosmetic and functional results similar to those of suturing with thread and also several advantages, such as reduction in perineal repair time and perineal pain intensity, exemption from the need for local anaesthesia, and more satisfaction among women [11].

A literature search showed the use of surgical glue in the perineal repair of first-degree tears and perineal skin in second-degree tears. Still, it remained a lack of knowledge in Obstetrics related to the effectiveness of the perineal repair of all tissue layers in second-degree tears and episiotomy [12]. In addition, it is essential to compare several types of surgical glues with other existing methods for perineal repair concerning perineal pain intensity, the long-term perineal healing process, the procedure duration, and the postpartum infection rates.

The study aimed to evaluate the effectiveness of surgical glue compared with standard suture thread in repairing first- and second-degree perineal tears and episiotomy in vaginal births concerning perineal pain and the healing process.

## Methods

### Design

A parallel randomised controlled open trial.

### Setting

The study was conducted at the birth centre of a municipal emergency and maternity hospital in the metropolitan region of São Paulo (Brazil), which assists women with low-risk full-term pregnancies.

### Participants and sample size

The population consisted of women with first- or second-degree spontaneous perineal tears or episiotomy. After delivery, this population was allocated into two experimental groups (EG) and two control groups (CG). The EG consisted of EG1: women who underwent repair of first-degree tears with glue, and EG2: women who underwent repair of second-degree tears or episiotomy with glue. The CG were as follows: CG1: women who underwent repair of first-degree tears with polyglactin 910 thread; and CG2: women who underwent repair of second-degree tears or episiotomy with polyglactin 910 thread.

The Bioestat® 5.3 software was used to estimate the sample size. The sample was constituted to detect a significant minimum difference of 2 points in the pain score between both perineal repair methods. *A priori*, a residual standard deviation of 3 points, a 5% alpha error and 80% test power, were considered. It resulted in a minimum sample of 35 parturient women in each group. Thus, the sample consisted of 140 women: 70 allocated to the EGs (EG1: *n* = 35; EG2: *n* = 35) and another 70 to the CGs (CG1: *n* = 35; CG2: *n* = 35).

### Inclusion criteria

The eligibility criteria were as follows: no previous vaginal birth; having up to 6 cm of cervical dilation at the time the woman was invited to participate in the research; not using steroid substances; not presenting leukorrhea or any signs of infection at the repair site; no difficulty understanding the Portuguese language or in communication; accepting to be subjected to perineal repair methods with surgical glue or suture thread.

The women included in the study underwent vaginal birth with first- and second-degree spontaneous perineal tears or episiotomy.

### Randomisation

The sequence for inclusion of the parturient in each group was randomised through an electronically-produced table of random numbers using the *Statistical Package for the Social Sciences* (SPSS) statistical program.

Opaque envelopes were employed, which were only opened at the perineal repair moment and contained the allocation to the glue or thread repair groups. One of the researchers was in charge of opening the envelopes.

### Interventions and materials

The interventions used surgical glue or suture thread to repair first- and second-degree perineal tears or episiotomy.

N-butyl-2-cyanoacrylate (Glubran-2®) is a synthetic surgical glue to be used on internal and external tissue, registered at the National Health Surveillance Agency (*Agência Nacional de Vigilância Sanitária*, ANVISA) under No. 80159010003. In contact with living tissue or a humid environment, the glue polymerises quickly, creating both an antiseptic barrier and a thin elastic film with high tensile strength, which ensures solid tissue adhesion that is not damaged by blood or organic fluids.

Proper glue application leads to solidification that starts in 1–2 s, finishing its reaction after nearly 60–90 s. In typical surgical procedures, the glue film is removed via hydrolytic degradation.

The polyglactin 910 thread consists of polyglycolic, synthetic and absorbable acid, which is fully absorbed in approximately 35 days via hydrolysis. The thread used for this study was a Vicryl rapid® 2.0 fast absorption thread with a continuous suture technique for perineal repair.

The polyglactin 910 thread consists of polyglycolic, synthetic and absorbable acid and is fully absorbed in approximately 35 days via hydrolysis.

The procedure described by Caroci-Becker et al. (2021 [13] was used to apply the Glubran-2® glue. It is worth noting that the woman was subjected to a new repair process with the same material in case of failure in perineal repair with surgical glue. The new repair procedure was performed with suture thread only in case of impossibility to repair with surgical glue due to bleeding, for instance.

For the suture with the Vicryl rapid® thread, local anaesthesia was applied with lidocaine 2% without vasoconstrictor. The perineal repair procedure was performed with thread using the non-anchored continuous technique in all the tissue layers.

### Outcomes

Pain occurrence and intensity were the primary outcomes evaluated, whereas the secondary outcome was perineal healing. The perineal repair time was also evaluated.

### Training of the team and pilot study

In order to improve the technique of applying the Glubran-2® glue, a training session was conducted with the researchers before data collection, in which the surgical glue was applied to beef tongue and other pieces of beef. After training the researchers, a case-series study was conducted [13] to implement the necessary adjustments to develop the current study.

### Data collection and measurements

The data were collected from March 2017 to September 2018 in six stages: stage 1: during labour and up to 2 h after the perineal repair procedure; stage 2: from 12 to 24 h postpartum; stage 3: from 36 to 48 h; stage 4: from 10 to 20 days; stage 5: from 50 to 70 days; and stage 6: from 6 to 8 months.

A form for the interview and data recording was explicitly developed for this research, which contained the following baseline characteristics: maternal age, ethnicity, schooling level, occupation, marital status, nutritional status, parity, gestational age, body mass index (BMI), newborn weight, and the outcomes variables. A pre-test was conducted to evaluate the form and the procedures that would be done during data collection.

As a first step, the researchers presented the study to professionals working in the service in order for them to accept, collaborate and integrate themselves into the research. During the recruitment, the researchers visited the study locus daily to locate the women who met the study's eligibility and inclusion criteria. The eligible women were invited to participate in the study when hospitalised.

Aiming to avoid bias in the data, the classification of the perineal trauma and the evaluation regarding the need for the repair procedure was in charge by the nurse-midwives of the birth centre, who were not part of the research team. Nevertheless, the nurse-midwives of the research team were in charge of the perineal repair procedure. Both groups used a digital stopwatch to measure the perineal repair time.

The professionals were asked to prescribe analgesics or anti-inflammatory medications if the puerperal women complained about pain so that perineal pain intensity could be better assessed. The participating women were instructed to request pain medications anytime they needed them. A medical evaluation was requested in case of complications related to the perineal repair procedure in the women from any research group.

In order to assess perineal pain intensity, the women were handed in the Visual Numeric Scale (VNS) to visualise and indicate the number corresponding to pain intensity. VNS consists of a horizontal line with values expressed in centimetres from 0 to 10, where zero is the total absence of pain and ten represents the worst pain possible [14]. This evaluation was performed 2 h postpartum to ensure a proper pain assessment between the groups, avoiding the anaesthesia bias in the suture group and all the other study stages.

The perineum healing process was evaluated using the REEDA scale in stages 1 and 4 of the study. The scale is indicated to evaluate the tissue recovery process after perineal trauma through five healing items: *redness, oedema, ecchymosis, discharge, and approximation* (coaptation of the wound edges) [15]. Each item evaluated was assigned a score from 0 to 3, where the maximum score (15) corresponds to the worst possible perineum healing result [16].

Each item evaluated was assigned a score from 0 to 3, where the maximum score (15) corresponds to the worst possible perineum healing result [16].

A Peri-Rule® ruler was used to measure hyperemia, oedema, ecchymosis and coaptation of the edges [17]. This ruler was wrapped in polyvinyl chloride (PVC) film and reused after cleaning with soap and water, followed by antisepsis with 70% alcohol.

In addition to the items on the REEDA scale, the researchers evaluated any other tissue damage or morbidity related to perineal repairs, such as hematoma, itching, wound infection, or allergic reaction.

Given the nature of the interventions and outcomes, there was no possibility of blinding, as both the women and the researchers were aware of the type of perineal repair performed and because, in the evaluation of the healing process, it is possible to see whether glue or suture thread was used.

### Statistical analysis

The data were double-typed into Epi-Info 6, and the database was validated and imported into Excel.

The mean and standard deviation (SD) were calculated for the descriptive analysis of the continuous quantitative variables. The Student's t-test was used to determine whether there was a statistical difference between the means of the two groups and analysis of variance (ANOVA) with the coefficient of determination for the multiple comparisons of means.

Absolute and relative frequencies were calculated for the categorical variables. The test used in the inferential analysis was Pearson's chi-square, and the approximate chi-square test in the Monte Carlo simulation was used in cross-tabulation.

In the longitudinal analysis, the generalised linear model (GLM) was employed, with Wald's chi-square test and analysis of the interactions of the effects (group and time or group and tear degree) based on linearly independent pair comparisons between estimated marginal means.

The significance level adopted was *p* ≤ 0.05. The analyses were performed in the following statistical packages: SAS System for Windows V8, SPSS for Windows (version 12.0) and Minitab Statistical Software – Release 13.1.

### Ethics

The project was approved by the Research Ethics Committee of the Arts, Sciences and Humanities School of the University of São Paulo—CAAE 44,832,615.1.0000.5390 and guaranteed the participants' rights. The study was registered in the Brazilian Registry of Clinical Trials, with registration data on 01/25/2018; last approval date on 01/25/2018; UTN code U1111-1184–2507; (www.ensaiosclinicos.gov.br/rg/RBR-2q5wy8). It is worth noting that the researchers are not linked to the manufacturers or distributors of the materials used in this study.

The study was registered in the Brazilian Registry of Clinical Trials, 01/25/2018; last approval date 01/25/2018; UTN code U1111-1184–2507; (www.ensaiosclinicos.gov.br/rg/RBR-2q5wy8). It is worth noting that the researchers are not linked to the manufacturers or distributors of the materials used in this study.

## Results

**Fig. 1:**
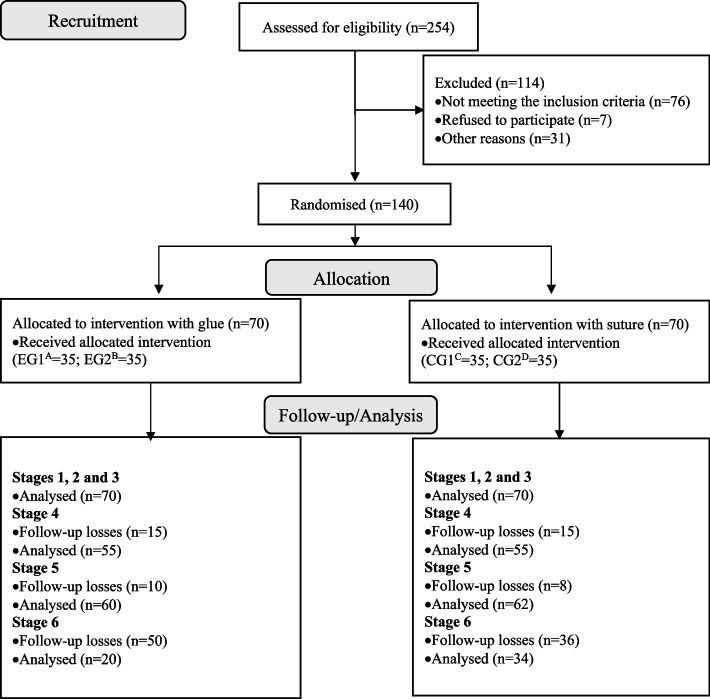
Flowchart corresponding to the participants. ^A^EG1: Experimental group 1; ^B^EG2: Experimental group 2; ^C^CG1: Control group 1;.^D^CG1: Control group 2

A total of 254 women met the eligibility criteria. Among these, 114 were excluded for the following reasons: not meeting the inclusion criteria (*n* = 76; caesarean section indicated during labour = 55; intact perineum = 21); refusing to participate (*n* = 7); other reasons (*n* = 31; first-degree tear when the number of EG participants was completed = 12; included in the pilot study = 19). Consequently, 140 women were included and randomised in the allocations: EG1 (*n* = 35), EG2 (*n* = 35), CG1 (*n* = 35), and CG2 (*n* = 35) groups, according to the type of trauma and the repair procedure performed (Fig. 1).

Among the 140 women who participated in the first three stages, 110 (78.6%) returned between 10 and 20 days postpartum (stage 4), 122 (87.1%) did so between 50 and 70 days (stage 5) and 54 (38.6%), between 6 and 8 months after delivery (stage 6). 30 (21.4%) women were follow-up losses between stages 3 and 4, 18 (12.9%) between stages 3 and 5, and 86 (61.4%) between stages 3 and 6 (Fig. 1).

The follow-up losses among the women were due to the following reasons: reported feeling good, waiving re-evaluation (*n* = 39) (stage 4: *n* = 10, 33.4%; stage 5: *n* = 5, 3.6%; stage 6: *n* = 24, 17.1%); did not answer or return the calls (*n* = 35) (stage 4: *n* = 4, 13.3%; stage 5: *n* = 4, 2.9%; stage 6: *n* = 27, 19.3%); did not attend the scheduled return visit or did not accept a home visit, without stating the reason (*n* = 25) (stage 4: *n* = 6, 20.0%; stage 5: *n* = 5, 3.6%; stage 6: *n* = 14, 10.0%); changed place of residence (*n* = 17) (stage 4: *n* = 3, 10.0%; stage 5: *n* = 2, 4.0%; stage 6: *n* = 12, 8.6%); home visit cancelled due to living in a highly hazardous location or requested at an inappropriate time (*n* = 14) (stage 4: *n* = 3, 10.0%; stage 5: *n* = 2, 1.4%; stage 6: *n* = 9, 6.4%); returned for consultation after the established period (stage 4: *n* = 4, 13.3%).

All the women enrolled on this study were nulliparous. There was no significant difference between the EGs and CGs (EG1, EG2, CG1, and CG2) concerning the sociodemographic and clinical characteristics (Table 1).

**Table 1:** Sociodemographic and clinical characteristics

Characteristics	All		Experimental Group (EG)						Control Group (CG)						*p*-value
			EG1^A^		EG2^B^		Total EG		CG1^C^		CG2^D^		Total CG		
	*n* = 140	%	*n* = 35	%	*n* = 35	%	*n* = 70	%	*n* = 35	%	*n* = 35	%	*n* = 70	%	
Skin** colour**															
Brown	73	52.1	21	60.0	19	54.3	40	57.2	12	34.3	21	60.0	33	47.2	0.096*
White	44	31.4	9	25.7	14	40.0	23	32.8	15	42.9	6	17.1	21	30.0	
Black	20	14.3	3	8.6	2	5.7	5	7.2	7	20.0	8	22.9	15	21.4	
Asian	3	2.2	2	5.7	-	-	2	2.8	1	2.8	-	-	1	1.4	
**Schooling**															
Incomplete Elementary School	7	5.0	2	5.7	2	5.7	4	5.7	1	2.8	2	5.7	3	4.3	0.733**
Complete Elementary School and incomplete High School	41	29.3	8	22.9	10	28.6	18	25.7	13	37.2	10	28.6	23	32.8	
Complete High School and incomplete Higher Education	86	61.4	24	68.6	22	62.9	46	65.7	19	54.3	21	60.0	40	57.2	
Complete Higher Education	6	4.3	1	2.8	1	2.8	2	2.9	2	5.7	2	5.7	4	5.7	
**Occupation**															
House chores	75	53.6	22	62.8	16	45.7	38	54.3	17	48.6	20	57.1	37	52.9	0.889**
Paid work	45	32.1	8	22.8	15	42.9	23	32.9	12	34.3	10	28.6	22	31.4	
Student	20	14.3	5	14.4	4	11.4	9	12.8	6	17.1	5	14.3	11	15.7	
**Marital status**															
With a partner	108	77.2	25	71.4	28	80.0	53	75.7	30	85.7	25	71.4	55	78.6	0.687**
Without a partner	32	22.8	10	28.6	7	20.0	17	24.3	5	14.3	10	28.6	15	21.4	
**Nutritional status**															
Low weight	36	26.9	13	37.1	6	17.6	19	27.5	10	32.3	7	20.7	17	26.1	0.823**
Adequate weight	52	38.8	12	34.3	15	44.1	27	39.1	10	32.3	15	44.1	25	38.5	
Overweight	29	21.6	7	20.0	9	26.5	16	23.2	7	22.5	6	17.6	13	20.0	
Obesity	17	12.7	3	8.6	4	11.8	7	10.2	4	12.9	6	17.6	10	15.4	
	Mean (SD)		Mean (SD)		Mean (SD)		Mean (SD)		Mean (SD)		Mean (SD)		Mean (SD)		
**Maternal age (years old)**															
	21.6 (4.5)		21.1 (4.5)		21.6 (4.3)		21.3 (4.4)		21.6 (4.0)		22.3 (5.3)		21.9 (4.7)		0.438***
**Gestational age (weeks)**															
	38.5 (8.1)		35.9 (11.2)		38.2 (1.2)		37.5 (8.1)		39.2 (1.1)		38.4 (6.8)		38.7 (4.8)		0.267***
**Newborn weight**															
	3285.1 (409.8)		3294.4 (429.5)		3319.67 (378.4)		3307.1 (401.6)		3140.0 (381.0)		3393.0 (425.1)		3262.8 (419.8)		0.058 ***
**BMI (kg/m**^**2)**^															
	27.7 (4.3)		27.8 (4.1)		28.3 (4.3)		28.0 (4.2)		26.6 (4.3)		28.3 (4.3)		27.4 (4.3)		0.430***

^A^EG1: Experimental group 1; ^B^EG2: Experimental group 2; ^C^CG1: Control group 1; ^D^CG1: Control group 2

^*^Monte Carlo test;

^**^Chi-square test;

^***^ANOVA

**Fig. 2:**
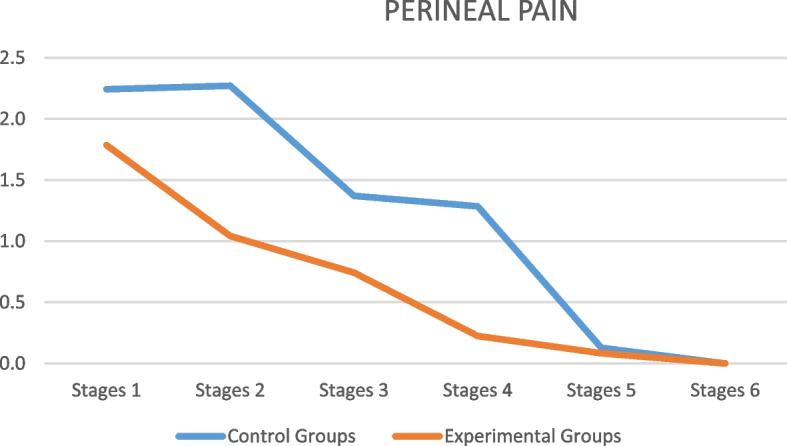
Perineal pain scores according to experimental and control groups along the trial stages. Wald's chi-square test: *p*-value ≤ 0.001 (pain score vs group); *p*-value ≤ 0.001 (pain score vs stage); *p*-value = 0.002 (pain score *vs* group vs stage)

Perineal pain intensity was evaluated in both types of perineal repair, from stage 1 to stage 6, verifying that perineal pain intensity was lower in the EGs (*p* ≤ 0.001), with a decrease in pain over time (*p* ≤ 0.001) (Fig. 2).

**Fig. 3:**
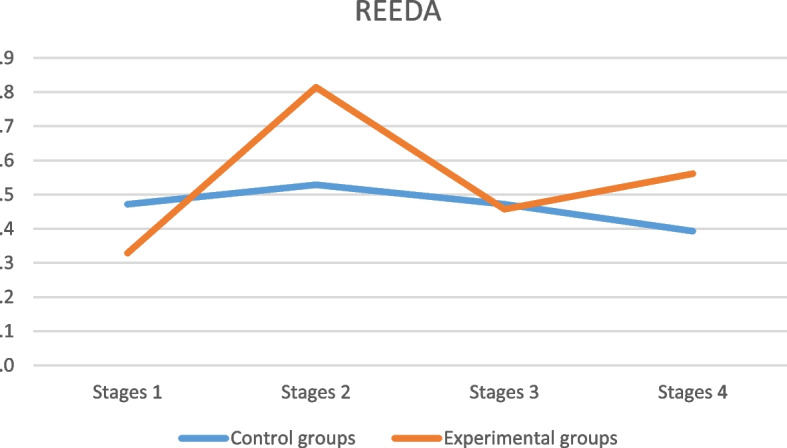
REEDA scores according to experimental and control groups along the trial stages. Wald's chi-square test: *p*-value = 0.464 (REEDA vs group); *p*-value ≤ 0.001 (REEDA vs stage); *p*-value = 0.006 (REEDA vs group vs stage)

The healing process according to groups is shown in Fig. 3.

The separate analysis of the REEDA scale items in the EGs and CGs presented a variation in the scores of the "edge approximation" item, observed with the group, to the study stage and the tear degree. Approximation was better among the women with first-degree tears who had perineal repair with suture (for the group and tear degree effects: *p* ≤ 0.001).

Over time, approximation was also better in the CG women (for the group, and time *p* ≤ 0.001). It is worth noting that the lower the REEDA score, the better the healing process.

In the EG, a new repair procedure with surgical glue was necessary for six women (8.6%; EG1 = 2; EG2 = 4) between 12 and 48 h postpartum. It is worth mentioning that these women continued in the study.

No need for a new repair procedure was verified in any of the CG women.

The hyperemia (*p* = 0.359), oedema (*p* = 0.059), ecchymosis (*p* = 0.712), and discharge (*p* = 0.260) items did not present any statistical difference.

The studied groups did not observe other tissue damage or morbidity related to perineal repairs, such as hematoma, itching, wound infection, or allergic reaction.

The perineal repair time was lower in the EG compared to the CG, with a mean of 12.1 (SD = 12.4) minutes *vs* 18.2 (SD = 10.1). It is worth noting that the repair time was not recorded in 22 (31.4%) women from the EG and 9 (12.9%) from the CG (Table 2).

**Table 2:** Perineal repair time (in minutes) according to the experimental and control groups

Group	n	%	Mean	SD
Experimental (EG)	70	100	12.1	12.4
EG1	22	31.4	8.2	5.6
EG2	26	37.2	15.5	15.4
Missing	22	31.4		
Control (CG)	70	100	18.2	10.1
CG1	29	41.4	12.8	8.0
CG2	32	45.7	23.1	9.2
Missing	9	12.9		

## Discussion

The principal findings of this study were that the use of surgical glue for the perineal repair of first- and second-degree tears and episiotomy in all tissue planes (skin, mucosa, and muscle) proved to be as effective as the standard suture method. It showed less pain, shorter procedure time, and a similar healing process.

The strengths of the present study were the design of a clinical, controlled, and randomised trial, in which the researchers rigorously followed all the eligibility and inclusion criteria to minimise selection biases. Also, a surgical glue suitable for deep tissue layers, such as muscles, allowed it to be used in second-degree tears and episiotomy.

In addition, the follow-up for a more extended period (up to 8 months) allowed the evaluation of the healing process until its complete resolution.

Another strength was the development of the surgical glue application technique and training for the team that participated in the study, which will allow the future sharing of this method.

There was a good acceptance among the women to participate in the research, which can be considered a strong study point. This finding surprised the researchers, as it was believed that, for being a new procedure, most women would not accept participating in the research out of fear, but this was not the case. On the contrary, some women allocated to the control group requested that the glue be used. However, the importance of randomisation in the types of perineal repair was explained by not allowing changing the method used.

The weaknesses found in the current study were the extended data collection period due to the small number of deliveries per day at the research site and the high number of exclusions related to the indication of cesarean section or intact perineum.

Due to the rapid polymerisation of the surgical glue, there was also difficulty in using surgical glue in the presence of heavy bleeding. In some cases, it was necessary to use more than one surgical glue ampoule (0.5 ml) to repair the tear, increasing the cost of the procedure. Another problem observed in the EG was the need for a new repair with surgical glue between 24 and 48 h after the initial procedure, which did not occur with the CG group. On the other hand, it was also observed that one glue unit could be used for more than one woman, depending on the degree and extent of the perineal tear.

A significant limitation was the price of the products. The surgical glue had an excessive cost (R$ 350.00) compared to the suture thread (R$ 22.00) in this study. However, it is worth mentioning that some materials and medications were not used when performing the repair with surgical glue, such as anaesthetics for the procedure and using an analgesic schedule for perineal pain after delivery, and the shorter time spent by the health professional to perform it. Although there was an option of using more economical surgical glues for skin and mucosa repair, only the glue chosen in the research is registered at ANVISA with approval to be used in the innermost layer (muscle) of perineal trauma.

The favourable results with surgical glue for repairing first- and second-degree tears concerning perineal pain agree with the results from other studies. A study in women with second-degree tears compared three skin closure methods (glue, suture, and non-suture) and showed that the lowest perineal pain intensity was with surgical glue. Assessed with a 100 mm visual analogue scale, the mean pain in the second postpartum week was 3.0 with glue, 5.0 with suture and 7.0 with no suture (*p* = 0.02). This difference was no longer observed three months after delivery (*p* = 0.31) [18]. Other studies also confirm the positive findings of using glue [11, 19, 20].

A study with a sample of 135 women, aiming to compare the use of Histoacryl® glue with the Monosyb® suture thread to repair first-degree tears, was done. It showed that women repaired with surgical glue had less perineal pain intensity in all situations evaluated (at rest, when sitting, walking and urinating) than those with sutures in the first week after birth. Nevertheless, no difference in perineal pain was found at 30 days postpartum [8].

As for the healing process, evaluated by the REEDA scale, the groups were similar regarding hyperemia, oedema, ecchymosis and discharge. The difference in edge coaptation was due to a lower score in the CG than in the EG, and it occurred mainly among women with second-degree tears up to 10 days after delivery. Other clinical trials that compared the use of glue to suture for perineal skin repair showed no significant difference in any of the items of the REEDA scale [20, 11, 19, 18, 9].

Nonetheless, it is essential to point out that the coaptation of deeper tissue layers was not evaluated in these studies, which had different design than ours.

The need to perform a new perineal repair procedure was also observed in a study conducted with 61 women where surgical glue was used to close the cutaneous episiotomy [21]. The percentage of 3.3% (2 women) who had superficial wound dehiscence in the first 48 h after birth was lower than the 8.6% observed in the current study, likely due to the study researching solely the cutaneous layer repair.

Only the current study was the repair with surgical glue performed in all tissue layers affected (skin, mucosa and muscles), except for the anal sphincter muscles, as the women with third or fourth-grade tears were not included.

As for the perineal repair time, in the EG, it was 6.1 min shorter than in the CG, corroborating the results of other studies [22, 11, 20, 9, 10]. It is worth emphasising that these studies used surgical glue on the mucosa or perineal skin and that this study evaluated the repair time of all tissue planes. Reducing the duration of the perineal repair procedure is essential, as it can decrease infections due to the lower exposure of tissues to microorganisms in the environment and abbreviate discomforts for women [23].

The results of this study related to less pain for women and a shorter procedure time are auspicious reasons for clinicians and policymakers to change the practice of perineal repair. Nevertheless, the excessive cost of surgical glue compared to suture thread can be an important limiting factor for its use in the delivery care practice, especially in health systems that face challenges due to the increased costs of materials and equipment, as well as in developing countries with few available resources.

Therefore, future cost analysis research is suggested, comparing all materials, procedures involved, and time spent by the professional in performing the two types of perineal repair. It is also suggested that further studies be conducted with several types of glue available and application methods to find the materials and techniques that contribute to the best cost–benefit to women. In addition, another vital factor to be analysed is the woman's satisfaction with both types of perineal repair.

## Conclusion

The n-butyl-2-cyanoacrylate surgical glue (GLUBRAN-2®) has proved to be effective because it has similar or better results in pain intensity and healing process compared to continuous suture with polyglycolic thread (Vicryl rapid®) in the repair of first- and second-degree perineal tears in vaginal births. Perineal repair with surgical glue can be an alternative method to standard suturing.

## Data Availability

The datasets and analyses generated during the current study are available from the author upon reasonable request.

## Abbreviations

EG: Experimental groups
CG: Control groups
SPSS: Statistical Package for the Social Sciences
ANVISA: Agência Nacional de Vigilância Sanitária
BMI: Body mass index
VNS: Visual numeric scale
REEDA: Redness, edema, ecchymosis, discharge, and approximation
PVC: Polyvinyl chloride
SD: Standard deviation
ANOVA: Analysis of variance
GLM: Generalised linear model

## References

[1] Steen M, Diaz M (2018). Perineal trauma: a women's health and well-being issue. Br J Midwifery.

[2] Vasileva P, Strashilov S, Yordanov A (2019). Postoperative management of postpartum perineal tears. Wound Med.

[3] Jansson MH, Franzén K, Hiyoshi A, Tegerstedt G, Dahlgren H, Nilsson K (2020). Risk factors for perineal and vaginal tears in primiparous women: the prospective POPRACT-cohort study. BMC Pregnancy Childbirth.

[4] Aslam P, Rasul N, Mushtaq Q, Mumtaz A, Sheikh H. Incidence and risk factors for perineal trauma. PJMHS 2022;16(1). 10.53350/pjmhs22161348

[5] Ahlund S, Radestad I, Zwedberg S, Lindgren H (2019). Perineal pain the first year after childbirth and uptake of postpartum check-up- a Swedish cohort study. Midwifery.

[6] Okeahialam NA, Thakar R, Sultan AH (2021). Healing of disrupted perineal wounds after vaginal delivery: a poorly understood condition. Brit J Nurs.

[7] Khatri R, Jain B, Mhapankar S, Kumar S (2021). Comparative study of continuous method and interrupted method of episiotomy in terms of healing of the surgical wound. Clin J Obstet Gynecol.

[8] Dasrilsyah RA, Kalok A, Ng BK, Ali A, Teik Chew K, Lim PS (2020). Perineal skin tear repair following vaginal birth; skin adhesive versus conventional suture: a randomised controlled trial. J Obstetr Gynaecol.

[9] Teixeira TT, Caroci AS, Brunelli WS, Riesco ML (2020). Tissue adhesive to repair first-degree perineal tears: a pilot randomised controlled trial. Clin Exp Obstet Gynecol.

[10] Ochiai AM, Araújo NM, Moraes SDTA, Caroci-Becker A, Sparvoli LG, Teixeira TT, Carvalho RR (2021). The use of non-surgical glue to repair perineal first-degree lacerations in normal birth: a non-inferiority randomised trial. Women Birth.

[11] Feigenberg T, Maor-Sagie E, Zivi E, Abu-Dia M, Bem-Meir A, Sela HY, Ezra Y (2014). Using adhesive glue to repair first-degree perineal tears: a prospective randomised controlled trial. Bio Med Res Interl.

[12] Li Y, Yi H, Huang Q, Lu H, Wang A. Effect of tissue adhesives in repairing perineal trauma during childbirth: a systematic review and meta-analysis. J Clin Nurs. 2021 Oct 20. doi: 10.1111/jocn.16086. Epub ahead of print. PMID: 34672033 10.1111/jocn.16086 34672033

[13] Caroci-Becker A, Brunelli WS, Lima MOP, Mendes EPB, Ochiai AM, Riesco MLG (2021). Use or surgical glue to repair intrapartum perineal lacerations: a case series study. Acta Paul Enferm.

[14] Pain: clinical manual for nursing practice Pain: clinical manual for nursing practice by Margo McCaffery Alexander Beebe Mosby Yearbook UK. Nurs Stand. 1994;9(11):55. 10.7748/ns.9.11.55.s69. 10.7748/ns.9.11.55.s69 27527475

[15] Childs C, Sandy-Hodgetts K, Broad C, Cooper R, Manresa M, Verdú-Soriano J (2020). Risk, prevention and management of complications after vaginal and caesarean section birth, birth-related wounds. J Wound Care.

[16] Davidson N (1974). REEDA: evaluating postpartum healing. J Nurse Midwifery.

[17] Tohill S, Metcalfe A. Perineal tear assessment and the development of the Peri-Rule. Perineal Care: An International Issue. 2005. 87-97.

[18] Swenson CW, Low LK, Kowalk KM, Fenner DE (2019). Randomised trial of 3 techniques of perineal skin closure during second-degree perineal laceration repair. JMWH.

[19] Seijmonsbergen-Schermers AE, Sahami S, Lucas C, Jonge AD (2015). Non suturing or skin adhesives versus suturing of the perineal skin after childbirth: a systematic review. Birth.

[20] Chamariya S, Prasad M, Chauhan A (2016). Comparison of dermabond adhesive glue with skin suture for repair of episiotomy. Int J Reprod Contracept Obstet Gynecol.

[21] Atesli EE, Guven S, CimilliSenocak GN, GuvendagGuven ES (2020). Comparison of the aesthetic and functional efficacy of subcuticular running closure (3/0 rapid absorbable 910 polyglactin) with N-BUTYL cyanoacrylate in episiotomy repair. Clin Exp Obstet Gynecol.

[22] Mota R, Costa F, Amaral A, Oliveira F, Santos CC, Ayres-De CD (2009). Skin adhesive versus subcuticular suture for perineal skin repair after episiotomy: a randomised controlled trial. Acta Obstet Gynecol Scand.

[23] Guideline. Perineal trauma assessment, repair and safe practice. The Women's. The Royal Women’s Hospital. Victoria, Australia. 2020;1-13. https://thewomens.r.worldssl.net/iages/uploads/downloadable-records/clinical-guidelines/perineal-trauma-assessment-repair-and-safe-practice_280720.pdf.

